# Delayed renal dysfunction after total body irradiation in pediatric malignancies

**DOI:** 10.1093/jrr/rru041

**Published:** 2014-06-08

**Authors:** Miho Watanabe Nemoto, Koichi Isobe, Gentaro Togasaki, Aki Kanazawa, Marie Kurokawa, Makoto Saito, Rintaro Harada, Hiroyuki Kobayashi, Hisao Ito, Takashi Uno

**Affiliations:** 1 Diagnostic Radiology and Radiation Oncology, Graduate School of Medicine, Chiba University, 1–8–1 Inohana, Chuo-ku, Chiba, 260–8677, Japan; 2 Department of Radiology, Chiba University Hospital, Chiba, Japan; 3 Department of Radiology, Sakura Medical Center, Toho University, Chiba, Japan

**Keywords:** renal toxicity, long-term survivor, total body irradiation, pediatrics

## Abstract

The purpose of this study was to retrospectively evaluate the incidence of delayed renal dysfunction after total body irradiation (TBI) in long-term survivors of TBI/hematopoietic stem cell transplantation (HSCT). Between 1989 and 2006, 24 pediatric patients underwent TBI as part of the conditioning regimen for HSCT at Chiba University Hospital. Nine patients who survived for more than 5 years were enrolled in this study. No patient had any evidence of renal dysfunction prior to the transplant according to their baseline creatinine levels. The median age at the time of diagnosis was 6 years old (range: 1–17 years old). The follow-up period ranged from 79–170 months (median: 140 months). Renal dysfunction was assessed using the estimated glomerular filtration rate (eGFR). The TBI dose ranged from 8–12 Gy delivered in 3–6 fractions over 2–3 d. The patients were treated with linear accelerators in the supine position, and the radiation was delivered to isocentric right–left and left–right fields via the extended distance technique. The kidneys and the liver were not shielded except in one patient with a left adrenal neuroblastoma. No patient required hemodialysis. The eGFR of four patients (44.4%) progressively decreased. The remaining patients did not demonstrate any eGFR deterioration. Only one patient developed hypertension. By evaluating the changes in eGFR, renal dysfunction among long-term survivors of TBI/HSCT could be detected. Our results suggested that the TBI schedule of 12 Gy in 6 fractions over three consecutive days affects renal function.

## INTRODUCTION

The use of hematopoietic stem cell transplantation (HSCT) after intensive chemotherapy and total body irradiation (TBI) is a widely accepted therapeutic approach for a number of hematological conditions as well as various malignant or benign diseases. Improvements in patient management, conditioning regimens, and TBI techniques have led to longer patient survival and better quality of life for patients. As the number of long-term survivors of HSCT grows, the assessment of late complications becomes increasingly important, particularly in children with a greater chance of long-term survival. In long-term survivors of TBI/HSCT, various complications such as cataracts, pulmonary fibrosis, veno-occlusive disease, graft-versus-host disease (GVHD), endocrine dysfunction, cognitive impairment, secondary malignancy, and renal dysfunction have been reported [1, 2]. Post-transplant renal dysfunction is one of the major complications that adversely affect the quality of life of patients that undergo HSCT. While multiple causes for these injuries have been identified, the administration of TBI at the time of conditioning is a major cause of injury [3–6]. Late renal dysfunction is mainly attributable to radiation-induced nephropathy, which is characterized by increased serum creatinine levels, proteinuria, anemia and high blood pressure [7, 8].

Studies of HSCT patients have demonstrated an association between TBI and nephrotoxicity; however, most published reports have involved short follow-up periods [2–4, 9]. Therefore, the incidence of radiotherapy-associated renal injuries might have been underreported due to its long latency period [7]. The long latency period for clinical renal toxicity was highlighted in a study of 67 patients with peptic ulcers, but without pre-existing hypertension, who were treated with 20 Gy over 3 weeks using a field that encompassed the left kidney [10]. Of these 67 patients, 31 (46%) developed renal toxicities within 8–19 years of undergoing radiotherapy, including seven patients who developed fatal uremia or malignant hypertension. Hence, renal toxicity is of great clinical significance in long-term survivors of TBI/HSCT. The purpose of this study was to retrospectively elucidate the delayed renal dysfunction after TBI in long-term survivors of TBI/HSCT.

## MATERIALS AND METHODS

Between 1989 and 2006, 24 pediatric patients underwent TBI as part of the conditioning regimen for HSCT at Chiba University Hospital. Follow-up data on patients were obtained from the medical records with Institutional review board (IRB) approval. Nine patients who survived for more than 5 years were used as the subjects of this study. The patients' characteristics and diagnoses are described in Table 1. The predominant underlying diseases were acute lymphoblastic leukemia (ALL) and acute myeloid leukemia (AML). The most common preparative regimen was TBI and cyclophosphamide. No patient exhibited any evidence of renal dysfunction prior to transplantation, as determined from their baseline creatinine levels, except for one patient who had a left adrenal neuroblastoma, which was treated with tumor extirpation and 12 Gy of intraoperative radiotherapy before the TBI. The median age at the time of diagnosis was 6 years old (range: 1–17 years old), and the follow-up period ranged from 79–170 months in length (median: 140 months).

**Table 1.** Patient characteristics

Age at diagnosis (year)	
Median	6
Range	1–17
Follow-up time (months)	
Median	140
Range	79–170
Gender	
Male	7 (77.8%)
Female	2 (22.2%)
Diagnosis	
ALL	4 (44.4%)
AML	3 (33.3%)
Other^a^	2 (22.2%)
TBI dose (Gy)	
12 Gy/6 fr	6 (66.7%)
10 Gy/3–4 fr	2 (22.2%)
8 Gy/3 fr	1 (11.1%)
Donor type	
Matched sibling	2 (22.2%)
Matched unrelated	3 (33.3%)
Mismatched related	
Autologous	1 (11.1%)
NA^b^	1 (11.1%)
Conditioning chemotherapy	
Cy^c^	5 (55.6%)
Cy + VP-16	2 (22.2%)
Other	2 (22.2%)

^a^Other diagnoses include: aplastic anemia (*n* = 1) and neuroblastoma (*n* = 1). ^b^NA = not available, ^c^Cy = cyclophosphamide.

Renal dysfunction was assessed using the estimated glomerular filtration rate (eGFR). We used the formula developed by Schwartz *et al*. to calculate the eGFR [11]. Based on a statistical analysis of the data of 186 children, they produced the following formula, which allows a subject's GFR to be accurately estimated from their plasma creatinine levels and height: eGFR = k*L*/Pcr, where eGFR is expressed in ml/min/1.73 m^2^, *L* represents height in cm, Pcr is the plasma creatinine level in mg/dl, and k is a constant of proportionality that depends on age and sex. In pediatric patients (under 21 years of age), we defined renal dysfunction as displaying an eGFR that was lower than the lower limit of normal value [11]. For patients who were older than 21, we defined renal dysfunction as exhibiting an eGFR of < 90 ml/min/1.73 m^2^ that continued to decrease in accordance with the chronic kidney disease guidelines of the Japanese Society of Nephrology [12].

## RESULTS

### Patient characteristics

The TBI doses ranged from 8–12 Gy and were delivered in 3–6 fractions over 2–3 d. Treatment was delivered at a nominal dose rate of 200 cGy/min at 1 m. The patients were treated using linear accelerators in the supine position, and the radiation was delivered to isocentric right–left and left–right fields via the extended distance (4 m) technique. The kidneys and liver were not shielded, except in one case involving a patient with a left adrenal neuroblastoma. Cyclosporine or cyclosporine combined with methotrexate was given to each patient for GVHD prophylaxis. The majority of patients were exposed to potentially nephrotoxic drugs during the post-transplant period, namely cyclosporine, aminoglycosides, vancomycin, amphotericin, and/or antiviral drugs.

### Renal dysfunction

**Fig. 1.**
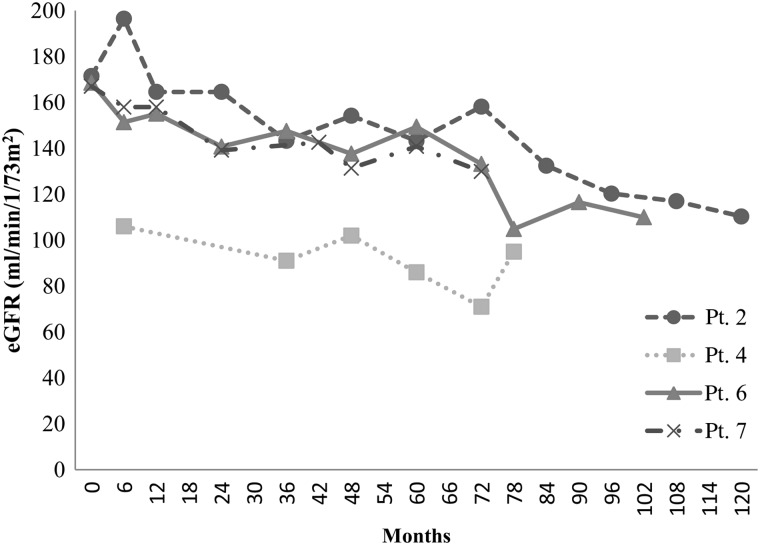
This graph shows the deterioration of eGFR in Patients (Pts) 2, 4, 6 and 7, who each received 12 Gy. The patients' eGFR progressively decreased.

**Fig. 2.**
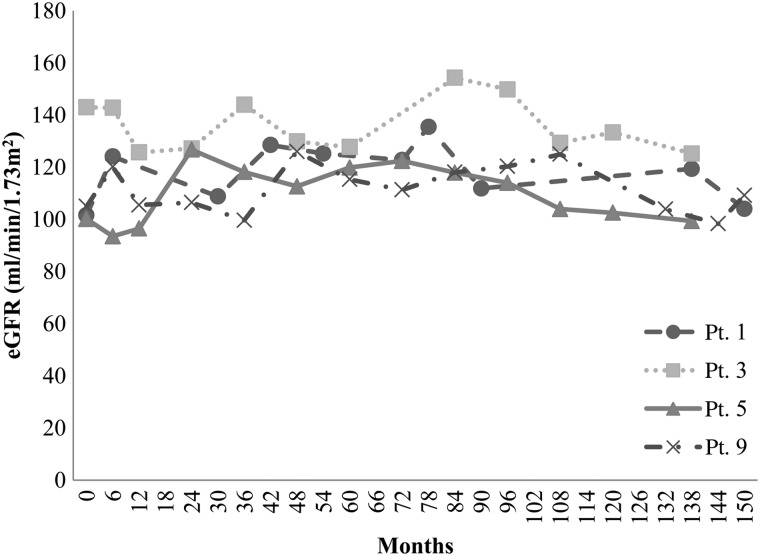
This graph shows the variation in eGFR over time in Pts 1, 3, 5 and 9, who received 8 Gy, 10 Gy, 12 Gy and 12 Gy, respectively. Five of the nine patients did not suffer any eGFR deterioration.

**Fig. 3.**
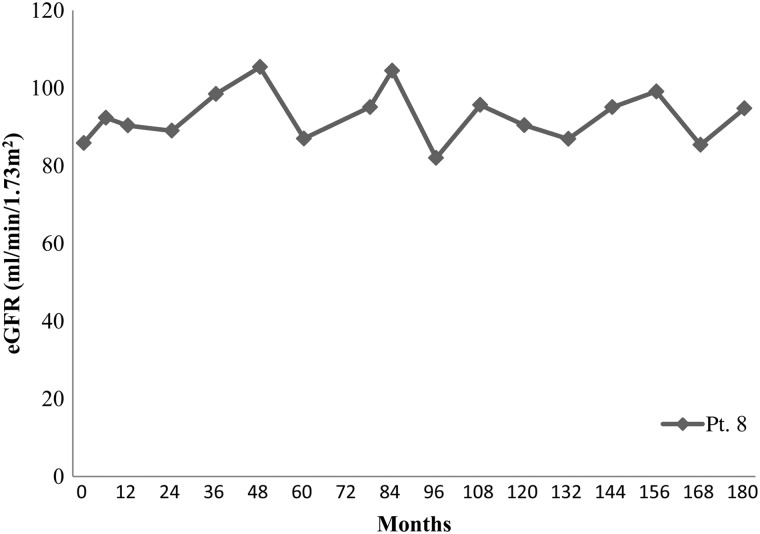
This graph shows the variation in eGFR over time in Pt. 8, in whom hypertension subsequently developed. The patient's eGFR was 80–100 ml/min/1.73 m^2^ and did not decline.

The patients' characteristics and renal function are described in Table 2. No patient required hemodialysis. Individual eGFR at TBI, and at two years and five years after TBI are shown in Table 3. The eGFR of four patients decreased progressively but remained within normal limits (Fig. 1). The remaining patients did not demonstrate any eGFR deterioration (Fig. 2). One patient developed hypertension. She had a left adrenal neuroblastoma, which was treated with tumor extirpation and 12 Gy of intraoperative radiotherapy followed by cisplatin-containing chemotherapy. Although her eGFR was fluctuated between 80 and 100 ml/min/1.73 m^2^, it did not decline (Fig. 3). Ultrasonography and renography revealed that the patient's left kidney was atrophic and had lost its function. Hypertension was detected 13 years after the TBI.

**Table 2.** Clinical data of nine patients

Patient No.	1	2	3	4	5	6	7	8	9
Age at diagnosis	1	6	3	15	17	16	15	2	6
Age at last exam.	12	17	13	26	23	23	22	16	18
Diagnosis	ALL	ALL	AA	AML	ALL	AML	AML	NB	ALL
TBI conditioning	8 Gy	12 Gy	10 Gy	12 Gy	12 Gy	12 Gy	12 Gy	10 Gy	12 Gy
Chemotherapy conditioning	Cy	Cy, VP-16	Cy	Cy	Cy	Cy, VP-16	Cy	Others	Others
Donor type	Matched unrelated	Matched sibling	Matched unrelated	Mismatched related	Mismatched related	Matched unrelated	NA	Auto	Matched sibling
Disease status	NED	NED	NED	NED	Relapse	NED	NED	NED	NED
Renal function		Decline		Decline		Decline	Decline	Hypertension	

ALL = acute lymphoblastic leukemia, AML = acute myeloid leukemia, AA = anaplastic anemia, NB = neuroblastoma, Cy = cyclophosphamide, VP-16 = etoposide, NED = no evidence of disease.

**Table 3.** eGFR of all patients at TBI and at two years and five years later

Patient	eGFR (ml/min/1.73 m^2^)		
	TBI	24 months	60 months
1	124.1	108.8	135.5
2	143	127.2	127.7
3	100.1	126.7	119.9
4	171.5	165.6	143.3
5	106	NA	101
6	169	141	149
7	167	139.2	140.8
8	85.9	89	87
9	105	106.5	115.3

Normal values of eGFR in children = 133 ± 27 ml/min/l.73 m^2^.

## DISCUSSION

Assessing the risk of treatment-related late renal dysfunction is very important, particularly in children. However, there are some difficult problems in assessing renal function. The definition of late renal toxicities varies between the studies, with the diagnosis involving elevated blood urea nitrogen, serum creatinine level [1, 9, 13], biopsy [2], kidney size [14], uremia [10], GFR [6] and eGFR [3]. In this study we used eGFR by Schwartz's formula in order to detect pre-clinical changes in renal function after TBI and to take height into account. In pediatric survivors, TBI/HSCT may lead to growth impairment due to endocrine dysfunction and impaired growth of bone. Recently, in the USA, the abbreviated Modification of Diet in Renal Disease (MDRD) equation [15] and Chronic Kidney Disease Epidemiology Collaboration (CKD-EPI) equation [16] are commonly used. Although these methods are more accurate than Schwartz's formula, they have not been validated in pediatric patients. Exposure to other multiple potentially nephrotoxic agents such as cyclosporine and antibiotics are another concern. Cheng *et al*. [17] suggested that pharmacotherapy has a major impact on kidney function and can modify the dose response. We were unable to ascertain how much each factor affected the incidence or severity of renal toxicities, as per previous similar studies. We consider that this issue needs to be clarified in further study.

The tolerance dose associated with a 5% risk of renal dysfunction at 5 years after unilateral whole kidney irradiation was estimated to be ∼23 Gy [18]. Nevertheless, a dose–response relationship between the renal radiation dose and the incidence of chronic renal dysfunction has never been demonstrated. The renal tolerance dose and the optimal schedule for fractionated TBI have also never been established. A recent extensive review by Kal *et al.* [19] reported the incidence of late renal dysfunction after TBI as a function of the biologically effective dose (BED). The tolerance dose for the kidneys during TBI was suggested to be ∼16 Gy (α/β = 2.5 Gy), which can be realized with fractionated TBI involving six fractions of 1.7 Gy. The total dose of 12 Gy that was prescribed for most of the patients in our study is higher than Kal's tolerance dose, suggesting that long-term survivors would develop late renal injuries following this administration. Most patients in our hospital received a total dose of 12 Gy in 6 fractions over three consecutive days, including all four patients with progressively decreasing eGFR. These findings suggest that this TBI schedule might reduce renal function, although it is difficult to reach definitive conclusions from such a small number of cases.

Some investigators who used selective renal shielding blocks have suggested that they reduced the risk of radiation-induced renal toxicities without decreasing the overall survival rate [3, 4, 20]. For example, it was reported that the use of selective renal-shielding blocks that restricted the renal dose to 10 Gy reduced the rate of renal dysfunction. Although the authors of the latter study concluded that the use of selective renal shielding did not appear to reduce the survival rate, such blocks might unintentionally decrease the dose delivered to the adjacent organs. In addition, TBI with selective renal-shielding blocks can be better performed with specific radiotherapy methods (such as anteroposterior and posteroanterior fields) using a moving-table technique, which is still uncommon in Japan.

The latency period for clinical renal toxicity has not been fully elucidated. It has been suggested that renal function stabilizes at one or two years after HSCT [21, 22]. Berg and Bolme did not observe any further deterioration of the eGFR after the first year after HSCT [21]. Patzer *et al.* reported that only two of 36 patients (6%) developed an eGFR of < 90 ml/min/1.73m^2^ in the 2 years after HSCT [22]. Half of their study patients were treated with autologous HSCT, which is less toxic than allogeneic HSCT. Frisk *et al*. measured renal function in children after autologous bone marrow transplantation [6]. A total of 26 patients had received TBI as part of their conditioning regimen. Seven patients in this group developed chronic renal impairment after bone marrow transplantation. Of all the patients, the lowest eGFR value was observed within 6 months of the bone marrow transplantation. After improving to some extent, the patient's eGFR stabilized. Although patients experienced some improvement in their eGFR, their latest values were below the normal limits. In our study, eGFR values of four patients progressively decreased, which suggests that the patients' renal function deteriorated over the long-term. This is comparable with the results of the above-mentioned peptic ulcer study. If the GFR continues to decrease, then patients might develop chronic kidney disease, which increases the risk of cardiovascular conditions such as stroke and myocardial infarction. Therefore, long-term follow-up is necessary for the assessment of late renal complications.

We should mention particularly the patient with a neuroblastoma, who developed hypertension 13 years after undergoing TBI. As well as the TBI, this case involved many additional nephrotoxic factors, e.g. the use of cisplatin and the administration of intraoperative irradiation to the left adrenal gland. In this case, the left kidney became atrophic and lost its function. It should be noted that this patient had a different background from the other patients, who had suffered only hematological conditions.

We should acknowledge the limitations of our study. We only evaluated a small number of patients. Given that only nine of 24 patients were assessed, there is a strong possibility of selection bias, with patients having severe or even fatal renal toxicities not being reported due to insufficient follow-up. In addition, our analysis was retrospective in nature. However, based on the extremely long follow-up time of this study (median, 140 months), our results suggested that the eGFR of four patients (who received 12 Gy) progressively decreased after TBI with an unexpectedly long latency. In addition, the majority of these patients underwent allogeneic transplant, which is by far the most concerning scenario for renal toxicity. Hence, a larger prospective follow-up study involving a pediatric hematologist is necessary in order to determine renal toxicity profiles and their clinical significance among long-term survivors of TBI/HSCT.
